# *Escherichia coli* O157 Outbreaks in the United States, 2003–2012

**DOI:** 10.3201/eid2108.141364

**Published:** 2015-08

**Authors:** Katherine E. Heiman, Rajal K. Mody, Shacara D. Johnson, Patricia M. Griffin, L. Hannah Gould

**Affiliations:** Centers for Disease Control and Prevention, Atlanta, Georgia, USA

**Keywords:** Escherichia coli O157, disease outbreaks, epidemiology, bacteria, United States, outbreaks, foodborne infections

## Abstract

Beef and leafy vegetables were the most common sources of these outbreaks.

## Medscape CME Activity

Medscape, LLC is pleased to provide online continuing medical education (CME) for this journal article, allowing clinicians the opportunity to earn CME credit. 

This activity has been planned and implemented in accordance with the Essential Areas and policies of the Accreditation Council for Continuing Medical Education through the joint providership of Medscape, LLC and Emerging Infectious Diseases. Medscape, LLC is accredited by the ACCME to provide continuing medical education for physicians.

Medscape, LLC designates this Journal-based CME activity for a maximum of 1.0 ***AMA PRA Category 1 Credit(s)^TM^***. Physicians should claim only the credit commensurate with the extent of their participation in the activity.

All other clinicians completing this activity will be issued a certificate of participation. To participate in this journal CME activity: (1) review the learning objectives and author disclosures; (2) study the education content; (3) take the post-test with a 75% minimum passing score and complete the evaluation at http://www.medscape.org/journal/eid; (4) view/print certificate.

**Release date: July 15, 2015; Expiration date: July 15, 2016**

## Learning Objectives

Upon completion of this activity, participants will be able to:

- Identify the most common source of outbreaks of *Escherichia coli* O157 in the United States
- Identify the most common specific food source associated with foodborne outbreaks of *E. coli* O157 in the United States
- Evaluate epidemiologic variables associated with the severity of *E. coli* O157 outbreaks
- Assess other epidemiologic data from outbreaks of *E. coli* O157 in the United States

## CME Editor

**Jean Michaels Jones,** BSN, Technical Writer/Editor,* Emerging Infectious Diseases. Disclosure: Jean Michaels Jones, BSN, has disclosed no relevant financial relationships.*

## CME Author

**Charles P. Vega, MD, **Clinical Professor of Family Medicine, University of California, Irvine. *Disclosure: Charles P. Vega, MD, has disclosed the following financial relationships: served as an advisor or consultant for McNeil Pharmaceuticals.*

## Authors

*Disclosures:* ***Katherine E. Heiman, MPH; Rajal K. Mody, MD, MPH; Shacara D. Johnson, MSPH; Patricia M. Griffin, MD;*** *and* ***L. Hannah Gould, PhD, MS, MBA,*** *have disclosed no relevant financial relationships.*
