# Correction: Vol. 21, No. 8

**DOI:** 10.3201/eid2401.C12401

**Published:** 2018-01

**Keywords:** errata, erratum, correction

An incorrect word in a sentence in *Escherichia coli* O157 Outbreaks in the United States, 2003–2012 (K.E. Heiman et al.) inadvertently changed the meaning. The sentence should have read, “The median annual number of outbreaks reported during 2008–2012 was higher than during 2003–2007 (45 vs. 33, p = 0.12) (Figure 1).” The article has been corrected online (https://wwwnc.cdc.gov/eid/article/21/8/14-1364_article).

