# Supplementary material for: Escherichia coli O157 Outbreaks in the United States, 2003–2012
Source: Emerg Infect Dis. 2015 Aug;21(8):1293–301. doi: 10.3201/eid2108.141364 (PMC4517704; doi:10.3201/eid2108.141364)
Supplement: Related texts

# Review article

## Abstract

Infections with the Shiga toxin–producing bacterium *Escherichia coli* O157 can cause severe illness and death. We summarized reported outbreaks of *E. coli* O157 infections in the United States during 2003–2012, including demographic characteristics of patients and epidemiologic findings by transmission mode and food category. We identified 390 outbreaks, which included 4,928 illnesses, 1,272 hospitalizations, and 33 deaths. Transmission was through food (255 outbreaks, 65%), person-to-person contact (39, 10%), indirect or direct contact with animals (39, 10%), and water (15, 4%); 42 (11%) had a different or unknown mode of transmission. Beef and leafy vegetables, combined, were the source of >25% of all reported *E. coli* outbreaks and of >40% of related illnesses. Outbreaks attributed to foods generally consumed raw caused higher hospitalization rates than those attributed to foods generally consumed cooked (35% vs. 28%). Most (87%) waterborne *E. coli* outbreaks occurred in states bordering the Mississippi River.

Signs and symptoms of infection with Shiga toxin–producing *Escherichia coli* O157 can include diarrhea that is often bloody, severe stomach cramps, and vomiting; infection can progress to hemolytic uremic syndrome (HUS) and death (*1*). In the United States, these infections and related illnesses are estimated to cost >$405 million annually (*2*).

*E. coli* O157 can be transmitted to humans through contaminated food and water, directly between persons, and through contact with animals or their environment. The most common reservoir is cattle, and ground beef is the most frequently identified vehicle of transmission to humans. *E. coli* O157 was first recognized as a foodborne pathogen after outbreaks during 1982 were linked to ground beef consumption (*1*). Since then, many other sources have been identified (*3*), mostly through outbreak investigations. We describe the epidemiology of *E. coli* O157 outbreaks during 2003–2012.

## Methods

### Surveillance

We identified *E. coli* O157 outbreaks in the United States using these sources: the Foodborne Disease Outbreak Surveillance System (FDOSS) for outbreaks during 2003–2012; the Waterborne Disease Outbreak Surveillance System (WBDOSS) for outbreaks during 2003–2010 (data were not available after 2010); the National Outbreak Reporting System (NORS) for outbreaks facilitated by transmission between persons, animal contact, environmental contamination, or with an unknown transmission mode during 2009–2012 (*4*); reports to the Centers for Disease Control and Prevention of person-to-person and animal contact–associated outbreaks during 2003–2008; and PubMed using these search terms: *Escherichia coli* O157 outbreak, STEC O157 outbreak, *Escherichia coli* O157, STEC O157, and O157. Data reported for each outbreak, with varying degrees of completeness, included primary transmission mode; states where exposure occurred; number of illnesses (culture-confirmed and epidemiologically linked); hospitalizations; HUS cases (defined by physician diagnosis; no laboratory data were collected); deaths, age, and sex distribution of patients; month the outbreak began; and setting where the outbreak occurred or, for foodborne disease outbreaks, the place of food preparation. For foodborne disease outbreaks, the implicated food (if identified) was reported. For waterborne disease outbreaks, water source type (e.g., recreational, drinking, waste water) was reported.

### Definitions

An *E. coli* O157 outbreak was defined as an event during which ≥2 persons had culture-confirmed *E. coli* O157:H7 or O157:NM infection resulting from a common exposure. Epidemiologically linked, clinically compatible cases without laboratory confirmation were included in the case count. States within the contiguous United States were categorized as “north” if they were located entirely north of parallel 37 north latitude and “south” if entirely below this latitude; states crossing this line (California and Nevada) were excluded from some analyses. 

Implicated foods were classified into categories by using an existing classification scheme (*5*) with 2 modifications: fruits and nuts were classified separately and meat acquired by hunting was classified with other meat. Implicated foods were further classified by whether they were usually consumed cooked (beef, other meats, poultry) or raw (leafy vegetables, fruits, unpasteurized dairy, sprouts). Steak was considered tenderized if described as tenderized or injected. Settings were classified as home; restaurant (e.g., restaurant, delicatessen, or banquet facility); institution (e.g., childcare center, school, or prison); fair; petting zoo; recreational area (e.g., park or beach); and other (e.g., grocery store, commercial production facility, or farm animal show).

### Analysis

We summarized demographic and epidemiologic findings by transmission mode and food categories for which >3 outbreaks were reported. Two periods, 2003–2007 and 2008–2012, were evaluated to assess changes over time. Severity was assessed by calculating hospitalization (no. hospitalized/total no. ill) and HUS rates (no. physician–diagnosed HUS cases/total no. ill). Culture-confirmation rates (no. culture-confirmed/total no. ill) were also calculated. Analyses of settings were limited to outbreaks that occurred in a single setting type. For transmission modes other than food, data for age group, sex, and culture confirmation were not available before 2009. We compared the median outbreak size and state outbreak rates (outbreaks with exposure occurring in a single state/sum of state populations during 2003–2012, from U.S. Census Bureau intercensal estimates) using the Kruskal-Wallis test, and performed categorical analyses using χ^2^ and Fisher exact tests; 2-tailed p values <0.05 were considered significant.

## Results

During 2003–2012, a total of 390 *E. coli* O157 outbreaks (353 O157:H7, 15 O157:NM, 1 O157:H7 and O157:NM, and 22 O157 with an unknown H antigen) were reported. These outbreaks resulted in 4,928 illnesses, 1,272 (26% of illnesses) hospitalizations, 299 (6%) physician-diagnosed HUS cases, and 33 (0.7%) deaths (Table 1). The median outbreak size was 6 illnesses (range 2–238). Primary transmission modes were foodborne (255 outbreaks, 65%), animal contact (39, 10%), person-to-person (39, 10%), waterborne (15, 4%), and other or unknown (42, 11%). Foodborne disease outbreaks caused the most illnesses (3,667, 74%), hospitalizations (1,035, 81%), physician-diagnosed HUS cases (209, 70%), and deaths (25, 70%). During 2009–2012, when data for culture-confirmed infections were collected for all outbreaks, the proportions of transmission were unknown modes (76%), foodborne (69%), person-to-person (62%), and animal contact (40%).

**Table 1:** Characteristics of *Escherichia coli* O157 outbreaks by transmission mode and food category, United States, 2003–2012*

Transmission source	Outbreaks (% of all outbreaks)	Illnesses (median outbreak size)	Hospitalizations (% of all illnesses)	Physician-diagnosed HUS (% of all illnesses)	Deaths (% of all illnesses)
Food	255 (65)	3,667 (6)	1,035 (29)	209 (6)	25 (0.7)
Beef	78 (20)	1,144 (7)	316 (28)	67 (6)	5 (0.4)
Poultry†	1 (0)	60	5 (8)	0	0
Other meat	7 (2)	39 (4)	12 (31)	4 (10)	0
Dairy	16 (4)	140 (6)	52 (37)	22 (16)	0
Leafy vegetables	29 (7)	922 (16)	321 (35)	53 (6)	7 (0.8)
Fruits	6 (2)	57 (8)	20 (35)	5 (9)	6 (10.5)
Sprouts	3 (1)	35 (13)	4 (11)	0	0
Nuts	1 (0)	8	3 (38)	0	0
Other foods‡	29 (7)	580 (11)	123 (21)	24 (4)	0
Food unknown	85 (22)	682 (5)	179 (26)	32 (5)	10 (1.5)
Animal contact	39 (10)	552 (6)	127 (23)	51 (9)	2 (0.4)
Person-to-person	39 (10)	322 (5)	45 (14)	24 (7)	2 (0.6)
Water	15 (4)	154 (6)	NA	NA	1 (0.6)
Other or unknown	42 (10)	233 (4)	65 (28)	15 (6)	3 (1.3)
Total	390 (100)	4,928 (6)	1,272 (27§)	299 (6§)	33 (0.7)

*HUS, hemolytic uremic syndrome; NA, not available. †Poultry was thought to be cross-contaminated by ground beef http://www.ct.gov/dph/lib/dph/infectious_diseases/ctepinews/vol29no5.pdf. ‡Outbreaks transmitted from other foods were 2 each of: guacamole, pico de gallo, salsa, and potato salad; and 1 each of: cookie dough; alfalfa sprouts and iceberg lettuce; baked beans and unknown fruit; meatballs, steak and green salad; cantaloupe and hamburger; lamb and beef; lettuce and green grapes; lime and bean dip; macaroni; Mexican wheat snack; sandwich; seafood; vegetable-based salad; pepperoni; jerky; multiple foods. Agencies sometimes report >1 food vehicle when epidemiologic evidence cannot distinguish between them even when it is likely that only one was the source. §Excludes 154 illnesses from the denominator that were associated with waterborne disease outbreaks because no data was collected for hospitalization and physician-diagnosed HUS cases for this transmission mode.

The median annual number of outbreaks reported during 2008–2012 was lower than during 2003–2007 (45 vs. 33, p = 0.12) (Figure 1). The median annual number of foodborne disease outbreaks did not change, although the number attributed to dairy (11 vs. 5) and fruits (5 vs. 2) more than doubled. The median annual number of outbreaks attributed to other modes of transmission did not change.

**Figure 1:** Number of *Escherichia coli* O157 outbreaks by transmission mode and year (n = 390), United States, 2003–2012.

### Vehicles

Of the 255 foodborne disease outbreak reports, 170 (67%) implicated a specific food, of which 141 (83%) could be classified into a single category. The implicated categories were beef (78 outbreaks, 55%), leafy vegetables (29, 21%), dairy (16, 11%), fruits (6, 4%), other meats (7, 5%), sprouts (3, 2%), nuts (1, 1%), and poultry (1, 1%) (Table 1). The types of beef implicated were ground beef (54 outbreaks, 69% of beef-associated outbreaks) and steak (10, 14%). The types of steak, reported in 5 outbreaks, were sirloin (4 outbreaks) and filet mignon (1). Steaks were mechanically tenderized in 5 outbreaks (2 sirloin, 1 filet mignon, 2 unknown). Other implicated meats were venison (3 outbreaks), lamb (2), and bison/buffalo (2).

In all 16 outbreaks attributed to dairy, unpasteurized products were implicated; 13 (81%) to unpasteurized milk and 3 (9%) to cheese made from unpasteurized milk. Most outbreaks attributed to leafy vegetables implicated lettuce (22 outbreaks, 76%), including romaine (3), iceberg (1), and mesclun mix (1); others in this category were spinach (4, 13%) and salads with unspecified types of greens (3,10%). Outbreaks attributed to fruit implicated unpasteurized apple cider (4 outbreaks), fruit salad (1), and strawberries (1), a newly identified food vehicle for this pathogen. Other newly identified food vehicles were hazelnuts sold in the shell (1 outbreak), packaged cookie dough (1), salsa (2), and pico de gallo (2). A total of 39 outbreaks were transmitted by contact with animals; >1 animal type was reported for 24 (62%), including cattle (15), goats (12), sheep (8), pigs (3), rabbits (3), chickens (2), and 1 each of elk, llama, alpaca, hedgehog, and dog. Every outbreak that identified a reported animal type included >1 ruminant animal. Although no specific animal types were listed for the remaining 14 outbreaks, livestock were reported for 2 and petting zoos or county fairs for 11.

Of the 15 waterborne disease outbreaks, 10 were attributed to recreational water (3 treated, 7 untreated), 3 to drinking water, and 1 possibly to wastewater. The water source was unknown for the remaining outbreak.

### Severity

The severity of outbreaks differed by transmission mode and among foodborne disease outbreaks by food category (Table 1). Hospitalization rates were lower for person-to-person outbreaks compared with all other transmission modes (14% vs. 28%, p<0.0001). Physician-diagnosed HUS rates were higher for animal contact outbreaks than for all other modes (9% vs. 6%, p = 0.0005). The 33 deaths occurred in foodborne (25 deaths), person-to-person (2), animal contact (2), and waterborne (1) disease outbreaks; transmission mode was unknown for 3 deaths.

Among foodborne disease outbreaks, hospitalization rates were lower for outbreaks attributed to beef than for all other food vehicles (28% vs. 35%, p<0.001). Hospitalization rates were higher among outbreaks attributed to foods generally consumed raw than for cooked foods (35% vs. 28%, p = 0.0001). Deaths occurred most commonly in outbreaks attributed to leafy vegetables (7 deaths, 0.8% of illnesses in leafy vegetable outbreaks), fruits (6, 11%), and beef (5, 0.4%).

### Demographic Characteristics

Age group distribution of cases varied by transmission mode and food category (Figure 2). Patient age group was available for 2,884 (81%) illnesses in foodborne outbreaks, 63 (90%) illnesses in waterborne outbreaks, and, during 2009–2012, 121 (96%) illnesses in person-to-person outbreaks and 189 (73%) illnesses in animal contact outbreaks. Most of the illnesses in person-to-person outbreaks (73, 60%) were among children <5 years old. The highest proportions of illnesses in waterborne and animal contact outbreaks (67% and 43%, respectively) were among persons 5–19 years of age. Foodborne disease occurred among persons in all age groups; only 8% of foodborne illnesses occurred among children <5 years of age, but >25% of dairy-associated illnesses were among children of this age group. The largest proportions of illnesses in outbreaks attributed to beef and dairy were among persons 5–19 years old (38% and 47%, respectively), whereas the largest proportion reported in outbreaks attributed to sprouts and leafy vegetables was among persons 20–49 years old (71% and 45%, respectively).

**Figure 2:** Percentage of *Escherichia coli* O157 outbreak illnesses by age group and A) transmission mode (n = 3,417) and B) selected food categories (n = 1,574), United States, 2003–2012

Overall, 55% of patients were women; the percentage did not vary significantly by transmission mode. Patient sex was available for 3,385 (92%) illnesses in foodborne disease outbreaks, 63 (90%) illnesses in waterborne outbreaks, and, from 2009–2012, 126 (97%) illnesses in person-to-person outbreaks and 193 (80%) illnesses in animal contact outbreaks. In foodborne disease outbreaks, the proportion of female patients was highest in outbreaks attributed to fruits (67%) and leafy vegetables (65%) and lowest in outbreaks attributed to meats other than beef (31%) (p<0.0001); cases were evenly distributed among women and men in outbreaks attributed to beef and dairy.

### Seasonality

*E. coli* O157 outbreaks occurred year-round; however, nearly half occurred during July–September (175, 46%) (Figure 3). The seasonality of foodborne outbreaks varied by food category. Beef-associated outbreaks occurred most often in July (21 outbreaks, 27%), and leafy vegetable-associated outbreaks in September (8, 28%). Waterborne disease outbreaks occurred most often during June (6, 40%) and outbreaks associated with animal contact during August (13, 37%).

**Figure 3:** Number of *Escherichia coli* O157 outbreaks by month and by A) transmission mode (n = 390) and B) selected food categories (n = 255), United States, 2003–2012.

### Geographic Distribution of Outbreaks and Multistate Outbreaks

Outbreaks were reported by 43 states (Figure 4). The median outbreak rate in northern states was >2× the rate in southern states (0.015 vs. 0.006/100,000 population, p = 0.005), which was also true for foodborne disease outbreaks (0.007 vs. 0.002, p = 0.02). Most waterborne disease outbreaks were reported in states bordering the Mississippi River (13, 87%), including all 3 drinking water outbreaks and all 7 recreational water outbreaks associated with lakes or other natural, untreated bodies of water. Waterborne disease outbreaks reported by California (1) and Idaho (1) were associated with treated recreational water.

**Figure 4:** Single-state *Escherichia coli* O157 outbreaks (n = 346) by state and transmission mode, United States, 2003–2012. A) Foodborne transmission (n = 211); B) animal contact transmission (n = 39); C) person-to-person transmission (n = 39); D) waterborne transmission (n = 15). Curved line denotes 37°N latitude.

Of the 390 outbreaks reported, 44 (17%), all foodborne, were multistate. Multistate outbreaks were larger than single state outbreaks (median 22 cases vs. 5, p<0.0001). The food categories implicated in multistate outbreaks were beef (22 outbreaks, 17 attributed to ground beef), leafy vegetables (12), unpasteurized dairy products (3), sprouts (1), nuts (1), and other meats (bison, 1).

### Settings

Among 222 foodborne outbreaks for which food preparation settings were reported, a single setting was reported for 183 (84%). Restaurants were the most common settings (73 outbreaks, 40%), followed by private homes (60, 33%) (Table 2). Beef was most frequently prepared in a home (34, 56%), followed by restaurants (15, 25%). Leafy vegetables were most often prepared in restaurants (13, 59%), followed by homes (4, 18%).

**Table 2:** *Escherichia coli* O157 outbreaks by setting (n = 357), United States, 2003–2012

Transmission source	Setting/no. outbreaks							
	Restaurant/ banquet facility	Home	Institutional*	Fair/petting zoo	Recreational area/facility†	Other‡	Unknown	Total
Food	73	60	16	2	0	32	39	222
Beef	15	34	3	0	0	9	8	69
Poultry	0	0	1	0	0	0	0	1
Other meat	1	5	0	0	0	0	0	6
Dairy	0	2	0	0	0	11	0	13
Leafy vegetables§	13	4	3	0	0	2	4	26
Fruits	0	2	0	1	0	2	0	5
Sprouts	1	0	0	0	0	0	0	1
Nuts	0	1	0	0	0	0	0	1
Other foods	13	6	0	0	0	2	3	24
Food unknown	30	6	9	1	0	6	24	76
Animal contact	0	1	2	17	0	4	15	39
Person-to-person	0	2	17	0	0	0	20	39
Water	0	2	2	0	7	4	0	15
Other or unknown	0	0	0	0	0	0	42	42
All outbreaks	73	65	37	19	7	38	116	357

*Institutional setting comprises camp, daycare, hospital, nursing home, prison, school, church, and workplace cafeteria. †Recreational area/facility comprises county and state park, beach, and a sports complex. ‡Other settings comprise a grocery store, commercial products, a 4H steer competition, communities, a farm, a National Western Stock Show, a public outdoor area, and a club. §Setting was not available for 3 outbreaks involving leafy vegetables.

Most (17, 89%) outbreaks with person-to-person transmission and a reported setting occurred in institutions; all except 1 institutional outbreak occurred in a childcare center. Petting zoos and fairs were the most common setting for outbreaks attributed to animal contact (17, 71%). Waterborne outbreaks occurred most often in recreational settings (7, 47%), including lakes (4), a pool (1), a sports complex (1), and an interactive fountain (1).

## Discussion

As in previous reports, our analysis found that food was the major mode of transmission for *E. coli* O157 outbreaks (*3*). Although beef, especially ground beef, remained the most common source (*3*), 14% of beef outbreaks were attributed to steak. Outbreaks attributed to foods generally consumed raw had higher hospitalization rates than other outbreaks, and leafy vegetables were the major source of outbreaks in the fall. Foodborne disease outbreak rates were highest in northern states, and most waterborne disease outbreaks occurred in states bordering the Mississippi River. New food vehicles were identified, including raw, prepackaged cookie dough (*6*), hazelnuts (*7*), strawberries (*8*), and salsa (*9*).

More outbreaks were reported during 2003–2012 than during the previous 20 years (*3*), although outbreaks were smaller (median 6 illnesses vs. 8). The hospitalization rate was higher than during the previous 20 years (27% vs. 17%), and the HUS rate was slightly higher (6% vs. 4%). Contaminated food was responsible for a larger percent of outbreaks during our study (65% vs. 52%), and the percent attributed to another or unknown mode decreased (10% vs. 21%). Among foodborne disease outbreaks, the percentage caused by *E. coli* O157 in beef decreased (31% vs. 47%), and among outbreaks associated with beef, the percentage linked to ground beef decreased (21% vs. 41%), whereas the percentage associated with other types of beef increased (9% vs. 6%). These differences might be partly attributable to continued improvements in surveillance, including the maturation of the national molecular subtyping network, PulseNet, which supported earlier detection of more outbreaks (*10*), as well as improved outbreak investigations and systematic, electronic reporting of outbreaks of all transmission modes (*4*).

Beef, particularly ground beef, continues to be the major source of *E. coli* O157 outbreaks, likely because cattle are the main reservoir for *E. coli* O157. Contamination of raw beef usually occurs during slaughter and meat processing by contact with hides contaminated with feces (*11*,*12*). Grinding can spread contamination through vast amounts of ground beef (*11*). Mechanically tenderizing steaks internalizes surface contamination (*13*,*14*), so steaks not cooked thoroughly could harbor *E. coli* O157. The risk for contamination varies for different locations on the carcass (*15*). Cattle carcasses sampled for microbial contamination had higher bacteria counts on sites on the rear (*16*,*17*).

Our finding that outbreaks attributed to leafy vegetables, dairy products, fruits, and other meats were more severe than outbreaks attributed to beef could have several explanations, including strain virulence and patient age and sex. Outbreak reports did not provide information on strain virulence factors. Outbreaks attributed to foods generally consumed raw had higher hospitalization rates than those attributed to cooked foods. Outbreaks with high proportions of illnesses among young children, especially those attributed to animal contact, dairy products, other meats, and fruits, also had the highest rates of physician-diagnosed HUS, which might relate to the higher risk for HUS among children (*18*). However, this finding was not true for person-to-person outbreaks, in which the proportion of illnesses among young children was highest. The reasons for this finding are unknown, but intensive case finding efforts during suspected person-to-person outbreak investigations might identify more mild illnesses and asymptomatic patients, decreasing the overall hospitalization rate. 

Higher hospitalization rates in outbreaks attributed to certain foods might be influenced by patient sex. For example, hospitalization rates were 35% for outbreaks associated with leafy vegetables, and 66% of illnesses were in female patients. Studies have found that women were at increased risk for HUS after *E. coli* O157 infection (*18*–*20*), although others have not found an association (*21*,*22*). Additionally, the proportion of illnesses in female patients might relate to gender-specific food preferences.

Most outbreaks occurred during the summer. Cattle shed the largest number of *E. coli* O157 organisms in their feces during summer months (*23*,*24*), coinciding with a higher prevalence of *E. coli* O157 on hides in processing plants (*25*). However, leafy vegetable-associated outbreaks exhibited distinct fall seasonality, which could be the result of summertime application to seedlings of irrigation water, soil amendments, or fertilizers that might contain more *E. coli* O157 organisms than other seasons. Therefore, leafy vegetables harvested during fall might be more likely to become contaminated than those grown at other times. Seasonality of leafy vegetable–associated outbreaks could also relate to harvest location. Nearly three quarters of leafy vegetable-associated outbreaks occurred during April–October; during this same time each year, most US-produced lettuce is harvested from the Salinas Valley (California) (*26*). For 4 of 5 recent outbreaks (2011–2012), detailed traceback information implicated lettuce harvested from farms in Salinas Valley (K.E. Heiman, unpub. data).

The cause of higher infection rates in northern states than southern states is unknown (*27*). Cattle density might play a role. In other countries, areas with the highest cattle density had some of the highest rates of human *E. coli* O157 infection (*28*). Cattle density does not appear to be higher in northern states compared with southern states (*25*), although it is very high in counties in California where most lettuce is produced (*29*). Other environmental factors might have a role. Edrington et al. found a stronger correlation between prevalence of *E. coli* O157 in cattle feces and longer day length than with higher ambient temperature, although both were statistically significant (*30*). However, several studies of prevalence of *E. coli* O157 in cattle feedlots have found either no variation by geography (*31*) or higher prevalence in herds in the south (*32*), where summertime daylight hours are shorter but ambient temperatures are higher. Geographic distribution might also be influenced by presence of cattle called “super shedders” that shed higher numbers of *E. coli* O157 bacteria (*33*). Differences in reporting by state could also affect geographic patterns (*4*).

Several factors might have contributed to our finding that waterborne outbreaks were reported primarily among states bordering the Mississippi River. Cattle density is high in the Midwest (*25*). *E. coli* O157-containing feces from cattle and other animals can be washed into nearby bodies of water, especially during heavy rainfall (*34*). Karst formations, which are rock formations that occur in several Midwest states, develop when acidic water begins to break down bedrock surfaces, allowing surface water to enter fractures in limestone and contaminate ground water; studies have shown that *E. coli* can survive in karst streams for prolonged periods (*35*). Karst formations exist in the counties that reported 2 of the 3 *E. coli* O157 outbreaks in which drinking water was the source; all were associated with well water. The remaining well water–associated outbreak occurred on a farm after heavy rain and flooding. However, karst formations are unlikely to explain outbreaks associated with untreated recreational water. Only 15 waterborne outbreaks were reported; therefore, interpretation of these findings is limited.

Our study had several limitations. Reported outbreaks likely underrepresent the number that occur (*36*). Many go unrecognized or unreported because they are not detected, no common source is found, or resources are not available for investigation (*37*). Sources of outbreak-related illnesses may differ from those of sporadic illnesses, although our findings were similar to those of another US study that examined risk factors for sporadic *E. coli* O157 infections (*38*). We analyzed outbreaks by their primary mode of transmission; however, outbreak-related infections could be transmitted by a variety of modes (e.g., several illnesses were transmitted from person-to-person in an outbreak linked to spinach). State and local health departments investigated and reported outbreaks; we did not verify transmission mode. Our findings might be influenced by changes in surveillance: outbreaks transmitted person-to-person, by animal contact, and by an unknown mode were not systematically reported until 2009 (*4*). We used physician-diagnosed HUS, which might overestimate the rate of HUS (*39*); however, the rate of physician-diagnosed HUS for all outbreak-related illnesses was 6%, the same as was reported in a study that used defined laboratory criteria (*2*). We found one third of foodborne disease outbreak reports did not have an identified implicated food item; therefore, the foods in this analysis might not be representative of all foodborne *E. coli* O157 outbreaks.

### Conclusions

More *E. coli* O157 outbreaks were reported during 2003–2012 than during the previous 20 years (*3*), probably because of continued improvements in surveillance. Outbreaks in the United States continue to be caused mostly by contaminated food, especially beef. People can protect themselves through proper handling of raw beef, by cooking nonintact beef products such as ground beef and mechanically tenderized steak to internal temperatures of at least 160°F, and by thorough handwashing after contact with animals or their environment. Clearly labeling mechanically tenderized beef might help consumers make safer choices. Pasteurizing dairy products and fruit juices is recommended. Further research is needed to identify interventions to reduce the risk of illness caused by consumption of raw produce. Ways must be found to encourage implementation of interventions such as decreasing contamination in the production of produce, vaccinating cattle, and irradiating foods (*40*). Consideration of the proximity of agriculture fields to cattle operations, as well as adherence to Good Agricultural Practices specified by the US Department of Agriculture (http://www.ams.usda.gov/AMSv1.0/gapghp) during production, will also help reduce risk. Continued surveillance is needed to monitor changes in food vehicles of contamination and to improve our understanding of the geographic distribution of outbreaks.

## References

[1] Riley LW, Remis RS, Helgerson SD, McGee HB, Wells JG, Davis BR, Hemorrhagic colitis associated with a rare *Escherichia coli* serotype. N Engl J Med. 1983;308:681–5. 10.1056/NEJM1983032430812036338386

[2] Frenzen PD, Drake A, Angulo FJ. Economic cost of illness due to *Escherichia coli* O157 infections in the United States. J Food Prot. 2005;68:2623–30 .1635583410.4315/0362-028x-68.12.2623

[3] Rangel JM, Sparling PH, Crowe C, Griffin PM, Swerdlow DL. Epidemiology of *Escherichia coli* O157:H7 outbreaks, United States, 1982–2002. Emerg Infect Dis. 2005;11:603–9. 10.3201/eid1104.04073915829201PMC3320345

[4] Hall AJ, Wikswo ME, Manikonda K, Roberts VA, Yoder JS, Gould LH. Acute gastroenteritis surveillance through the National Outbreak Reporting System, United States. Emerg Infect Dis. 2013;19:1305–9. 10.3201/eid1908.13048223876187PMC3739540

[5] Gould LH, Walsh KA, Vieira AR, Herman K, Williams IT, Hall AJ, Surveillance for foodborne disease outbreaks—United States, 1998–2008. MMWR Surveill Summ. 2013;62:1–34 .23804024

[6] Neil KP, Biggerstaff G, MacDonald JK, Trees E, Medus C, Musser KA, A novel vehicle for transmission of *Escherichia coli* O157:H7 to humans: multistate outbreak of E. coli O157:H7 infections associated with consumption of ready-to-bake commercial prepackaged cookie dough–United States, 2009. Clin Infect Dis. 2012;54:511–8. 10.1093/cid/cir83122157169

[7] Miller BD, Rigdon CE, Ball J, Rounds JM, Klos RF, Brennan BM, Use of traceback methods to confirm the source of a multistate *Escherichia coli* O157:H7 outbreak due to in-shell hazelnuts. J Food Prot. 2012;75:320–7. 10.4315/0362-028X.JFP-11-30922289593PMC13378396

[8] Laidler MR, Tourdjman M, Buser GL, Hostetler T, Repp KK, Leman R, *Escherichia coli* O157: H7 infections associated with consumption of locally grown strawberries contaminated by deer. Clin Infect Dis. 2013;57:1129–34. 10.1093/cid/cit46823876397

[9] Kendall ME, Mody RK, Mahon BE, Doyle MP, Herman KM, Tauxe RV. Emergence of salsa and guacamole as frequent vehicles of foodborne disease outbreaks in the United States, 1973–2008. Foodborne Pathog Dis. 2013;10:316–22. 10.1089/fpd.2012.132823461608

[10] Swaminathan B, Barrett TJ, Hunter SB, Tauxe RV. CDC PulseNet Task Force. PulseNet: the molecular subtyping network for foodborne bacterial disease surveillance, United States. Emerg Infect Dis. 2001;7:382–9. 10.3201/eid0703.01730311384513PMC2631779

[11] Erickson MC, Doyle MP. Food as a vehicle for transmission of Shiga toxin-producing *Escherichia coli.* J Food Prot. 2007;70:2426–49 .1796963110.4315/0362-028x-70.10.2426

[12] Hussein HS. Prevalence and pathogenicity of Shiga toxin–producing *Escherichia coli* in beef cattle and their products. J Anim Sci. 2007;85(Suppl):E63–72. 10.2527/jas.2006-42117060419

[13] Luchansky JB, Phebus RK, Thippareddi H, Call JE. Translocation of surface-inoculated *Escherichia coli* O157:H7 into beef subprimals following blade tenderization. J Food Prot. 2008;71:2190–7 .1904426010.4315/0362-028x-71.11.2190

[14] Laine ES, Scheftel JM, Boxrud DJ, Vought KJ, Danila RN, Elfering KM, Outbreak of *Escherichia coli* O157:H7 infections associated with nonintact blade-tenderized frozen steaks sold by door-to-door vendors. J Food Prot. 2005;68:1198–202 .1595470710.4315/0362-028x-68.6.1198

[15] Stopforth JD, Lopes M, Shultz JE, Miksch RR, Samadpour M. Microbiological status of fresh beef cuts. J Food Prot. 2006;69:1456–9 .1678687410.4315/0362-028x-69.6.1456

[16] Elder RO, Keen JE, Siragusa GR, Barkocy-Gallagher GA, Koohmaraie M, Laegreid WW. Correlation of enterohemorrhagic *Escherichia coli* O157 prevalence in feces, hides, and carcasses of beef cattle during processing. Proc Natl Acad Sci U S A. 2000;97:2999–3003. 10.1073/pnas.97.7.299910725380PMC16181

[17] Bell RG. Distribution and sources of microbial contamination on beef carcasses. J Appl Microbiol. 1997;82:292–300. 10.1046/j.1365-2672.1997.00356.x12455892

[18] Gould LH, Demma L, Jones TF, Hurd S, Vugia DJ, Smith K, Hemolytic uremic syndrome and death in persons with *Escherichia coli* O157: H7 infection, foodborne diseases active surveillance network sites, 2000–2006. Clin Infect Dis. 2009;49:1480–5. 10.1086/64462119827953

[19] Cimolai N, Carter JE, Morrison BJ, Anderson JD. Risk factors for the progression of *Escherichia coli* O157:H7 enteritis to hemolytic-uremic syndrome. J Pediatr. 1990;116:589–92. 10.1016/S0022-3476(05)81609-92181099

[20] Chang HG, Tserenpuntsag B, Kacica M, Smith PF, Morse DL. Hemolytic uremic syndrome incidence in New York. Emerg Infect Dis. 2004;10:928–31. 10.3201/eid1005.03045615200834PMC3323227

[21] Tserenpuntsag B, Chang HG, Smith PF, Morse DL. Hemolytic uremic syndrome risk and *Escherichia coli* O157:H7. Emerg Infect Dis. 2005;11:1955–7. 10.3201/eid1112.05060716485489PMC3367638

[22] Rowe PC, Orrbine E, Lior H, Wells GA, Yetisir E, Clulow M, Risk of hemolytic uremic syndrome after sporadic *Escherichia coli* O157:H7 infection: results of a Canadian collaborative study. J Pediatr. 1998;132:777–82. 10.1016/S0022-3476(98)70303-89602185

[23] Hancock DD, Besser TE, Rice DH, Herriott DE, Tarr PI. A longitudinal study of *Escherichia coli* O157 in fourteen cattle herds. Epidemiol Infect. 1997;118:193–5. 10.1017/S09502688960072129129597PMC2808780

[24] Dunn JR, Keen JE, Thompson RA. Prevalence of Shiga-toxigenic *Escherichia coli* O157:H7 in adult dairy cattle. J Am Vet Med Assoc. 2004;224:1151–8. 10.2460/javma.2004.224.115115074864

[25] Barkocy-Gallagher GA, Arthur TM, Rivera-Betancourt M, Nou X, Shackelford SD, Wheeler TL, Seasonal prevalence of Shiga toxin-producing *Escherichia coli*, including O157:H7 and non-O157 serotypes, and *Salmonella* in commercial beef processing plants. J Food Prot. 2003;66:1978–86 .1462727210.4315/0362-028x-66.11.1978

[26] Agricultural Marketing Resource Center. Lettuce Profile. 2005 4/2012 [cited 2013 May 29]; http://www.agmrc.org/commodities__products/vegetables/lettuce/

[27] Sodha SV, Heiman K, Gould LH, Bishop R, Iwamoto M, Swerdlow DL, National patterns of *Escherichia coli* O157 infections, USA, 1996–2011. Epidemiol Infect. 2014;143:267–73. 10.1017/S095026881400088024731294PMC6542353

[28] Frank C, Kapfhammer S, Werber D, Stark K, Held L. Cattle density and Shiga toxin–producing *Escherichia coli* infection in Germany: increased risk for most but not all serogroups. Vector Borne Zoonotic Dis. 2008;8:635–44. 10.1089/vbz.2007.023718402549

[29] United States Department of Agriculture. 2007 Census of Agriculture, United States, online census highlights, Ag Atlas Maps: USDA. 2009 [cited 2015 May 6]. http://agcensus.usda.gov/Publications/2007/Online_Highlights/Ag_Atlas_Maps/

[30] Edrington TS, Callaway TR, Ives SE, Engler MJ, Looper ML, Anderson RC, Seasonal shedding of *Escherichia coli* O157: H7 in ruminants: a new hypothesis. Foodborne Pathog Dis. 2006;3:413–21. 10.1089/fpd.2006.3.41317199523

[31] Hancock DD, Rice DH, Thomas LA, Dargatz DA, Besser TE. Epidemiology of *Escherichia coli* O157 in feedlot cattle. J Food Prot. 1997;60:462–5 http://www.ingentaconnect.com/content/iafp/jfp/1997/00000060/00000005/art00001.3119558110.4315/0362-028X-60.5.462

[32] Garber L, Wells S, Schroeder-Tucker L, Ferris K. Factors associated with fecal shedding of verotoxin-producing *Escherichia coli* O157 on dairy farms. J Food Prot. 1999;62:307–12 .1041920010.4315/0362-028x-62.4.307

[33] Chase-Topping M, Gally D, Low C, Matthews L, Woolhouse M. Super-shedding and the link between human infection and livestock carriage of *Escherichia coli* O157. Nat Rev Microbiol. 2008;6:904–12. 10.1038/nrmicro202919008890PMC5844465

[34] Cooley M, Carychao D, Crawford-Miksza L, Jay MT, Myers C, Rose C, Incidence and tracking of *Escherichia coli* O157:H7 in a major produce production region in California. PLoS ONE. 2007;2:e1159. 10.1371/journal.pone.000115918174909PMC2174234

[35] Davis RK, Hamilton S, Brahana JV. Escherichia coli survival in mantled Karst springs and streams, Northwest Arkansas Ozarks, USA. J Am Water Resour Assoc. 2005;41:1279–87. 10.1111/j.1752-1688.2005.tb03800.x

[36] Scallan E, Jones TF, Cronquist A, Thomas S, Frenzen P, Hoefer D, Factors associated with seeking medical care and submitting a stool sample in estimating the burden of foodborne illness. Foodborne Pathog Dis. 2006;3:432–8. 10.1089/fpd.2006.3.43217199525

[37] National Association of County and City Health Officials. Local health department job losses and program cuts: findings from January/February 2010 survey. Washington: The Association; 2010.

[38] Voetsch AC, Kennedy MH, Keene WE, Smith KE, Rabatsky-Ehr T, Zansky S, Risk factors for sporadic Shiga toxin–producing *Escherichia coli* O157 infections in FoodNet sites, 1999–2000. Epidemiol Infect. 2007;135:993–1000. 10.1017/S095026880600756417147834PMC2870643

[39] Ong KL, Apostal M, Comstock N, Hurd S, Webb TH, Mickelson S, Strategies for surveillance of pediatric hemolytic uremic syndrome: Foodborne Diseases Active Surveillance Network (FoodNet), 2000–2007. Clin Infect Dis. 2012;54(suppl 5):S424–31. 10.1093/cid/cis20822572665PMC3348948

[40] Doyle MP, Erickson MC. Opportunities for mitigating pathogen contamination during on-farm food production. Int J Food Microbiol. 2012;152:54–74. 10.1016/j.ijfoodmicro.2011.02.03721474196

# Review article

## Medscape CME Quiz

## Earning CME Credit

To obtain credit, you should first read the journal article. After reading the article, you should be able to answer the following, related, multiple-choice questions. To complete the questions (with a minimum 75% passing score) and earn continuing medical education (CME) credit, please go to http://www.medscape.org/journal/eid. Credit cannot be obtained for tests completed on paper, although you may use the worksheet below to keep a record of your answers. You must be a registered user on Medscape.org. If you are not registered on Medscape.org, please click on the “Register” link on the right hand side of the website to register. Only one answer is correct for each question. Once you successfully answer all post-test questions you will be able to view and/or print your certificate. For questions regarding the content of this activity, contact the accredited provider, CME@medscape.net. For technical assistance, contact CME@webmd.net. American Medical Association’s Physician’s Recognition Award (AMA PRA) credits are accepted in the US as evidence of participation in CME activities. For further information on this award, please refer to http://www.ama-assn.org/ama/pub/about-ama/awards/ama-physicians-recognition-award.page. The AMA has determined that physicians not licensed in the US who participate in this CME activity are eligible for *AMA PRA Category 1 Credits™*. Through agreements that the AMA has made with agencies in some countries, AMA PRA credit may be acceptable as evidence of participation in CME activities. If you are not licensed in the US, please complete the questions online, print the certificate and present it to your national medical association for review.

## Article Title:  *Escherichia coli* O157 Outbreaks in the United States, 2003–2012

## CME Questions

**1. You recently treated a 28-year-old man with a 3-day history of bloody diarrhea. His stool culture returns with positive results for *Escherichia coli* O157. In the current study by Heiman and colleagues, what was the most common source of outbreaks of *E. coli* O157?**A. FoodborneB. Person-to-personC. Animal contactD. Other/unknown**2. Which of the following specific foods was associated with the majority of foodborne cases of *E. coli* O157 in the current study?**A. Beef B. DairyC. Leafy vegetablesD. Poultry**3. You call the patient with the laboratory result, and he is feeling somewhat better since starting antibiotics. Which of the following statements regarding the severity of illness with *E. coli* O157 in the current study is most accurate?**A. Person-to-person outbreaks accounted for the majority of cases of mortalityB. Person-to-person outbreaks were associated with the highest rates of hospitalizationC. Hemolytic uremic syndrome was most associated with outbreaks from animal contactD. Outbreaks related to beef were associated with the highest rates of hospitalization**4. What else should you consider regarding the epidemiology of *E. coli* O157 outbreaks in the current study?**A. Nearly 80% of patients were menB. Most cases occurred in the winterC. Outbreaks were more common in Southern vs Northern statesD. More than 80% of waterborne outbreaks were reported in states that border the Mississippi River

## Activity Evaluation

**1:** The activity supported the learning objectives.

Strongly Disagree				Strongly Agree
1	2	3	4	5
**2. The material was organized clearly for learning to occur.**				
Strongly Disagree				Strongly Agree
1	2	3	4	5
**3. The content learned from this activity will impact my practice.**				
Strongly Disagree				Strongly Agree
1	2	3	4	5
**4. The activity was presented objectively and free of commercial bias.**				
Strongly Disagree				Strongly Agree
1	2	3	4	5
